# Right Transverse Testicular Ectopia: A Nonclassified Variant Confirmed on Laparoscopy

**DOI:** 10.1155/2021/4096762

**Published:** 2021-10-08

**Authors:** Landry Mbouché, E. Njuma Tamufor, K. G. Fossi, A. S. Salihou, D. E. C. Dikongue, Salihou Fadi, Bienvenu Binwe, F. F. Mouafo Tambo

**Affiliations:** ^1^Faculty of Medicine and Biomedical Sciences, University of Yaounde I, Cameroon; ^2^Yaounde Gynaeco-Obstetric and Paediatric Hospital, Cameroon

## Abstract

Transverse testicular ectopia is a rare anomaly characterized by testicular descent into the scrotum through the same inguinal canal. Here, we report the case of a 15-year-old boy diagnosed with transverse testicular ectopia wherein both testes descended through separate inguinal canals. He underwent a diagnostic laparoscopy which helped to identify both spermatic cords entering both inguinal canals separately. During scrotal exploration, both testes were found in the same side. Transseptal orchidopexy was performed. The short-term follow-up is uneventful.

## 1. Introduction

Ectopic testis is the migration of the testis away from its normal route of descent. The common sites for ectopic testes include the superficial inguinal pouch, femoral canal, perineum, pubopenile region, and opposite scrotum and is also called transverse testicular ectopia (TTE). The latter is a rare form of testicular ectopia wherein both testes descend along the same inguinal route and are found in the same hemiscrotum [1]. Following the first description by Von Lenhossek in 1886 [2], it has been reported in the literature in over a hundred cases as crossed testicular ectopia, pseudoduplication, unilateral double testes, transverse aberrant testicular maldescent, or transverse testicular ectopia [3] with patients' ages ranging from 3 days to 77 years [4]. These cases have been reported with various associated malformations. We present here the case of a 15-year-old boy with a transverse testicular ectopia. Laparoscopy and scrotal operation showed both spermatic cords entering their independent inguinal canals while the testes are located on the same side.

## 2. Case Presentation

**Figure 1:**
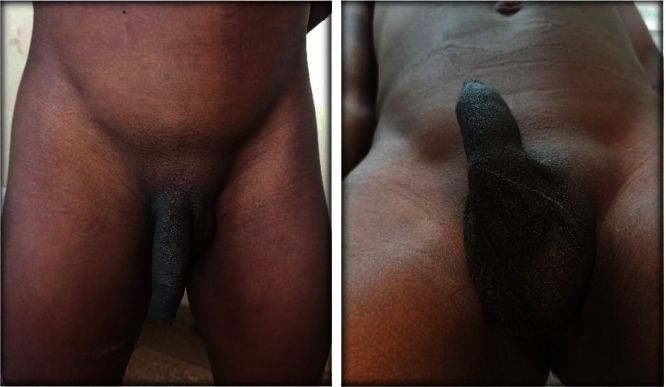
Patient in standing and lying positions showing hypoplasia of right hemiscrotum and left suprascrotal bulge.

**Figure 2:**
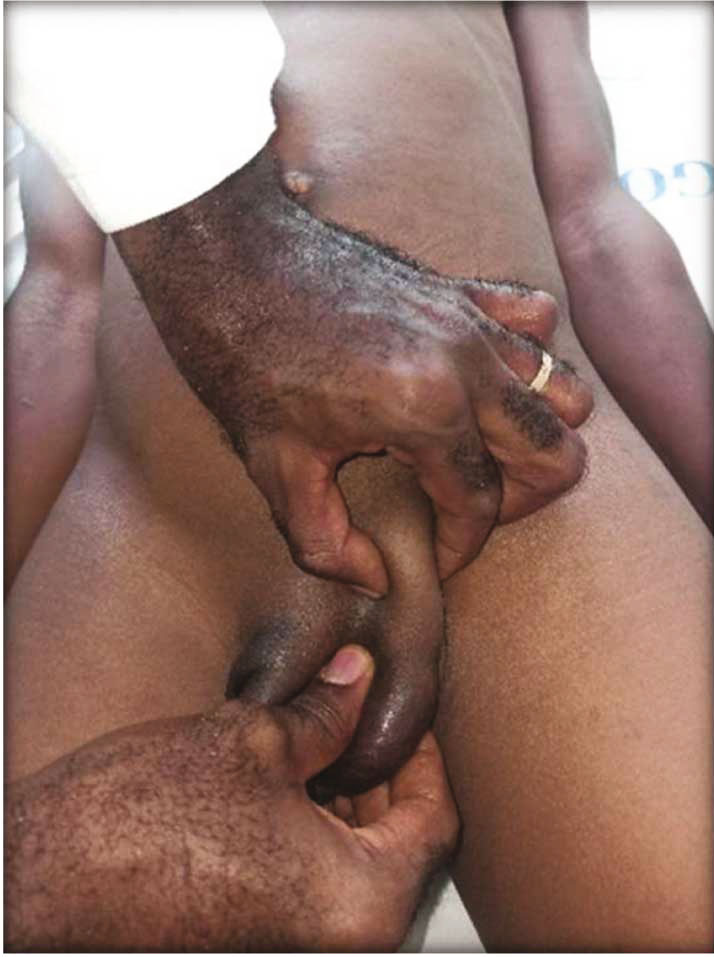
Examiner isolating two separate masses on the left path of testicular descent.

We received a 15-year-old male at our outpatient consultation with complaints of an abnormal looking scrotum and a left scrotal swelling. He denied testicular pain. His past history was notable for the presence of this scrotal anomaly from birth with several consultations done in infancy and no definitive diagnosis nor management proposed. On examination, there was a vacant, hypoplastic right hemiscrotum with no palpable right testis along its normal course; a normal looking left hemiscrotum containing a testis; an ovoid shaped mass at the left suprascrotal area which was firm, nontender and not fixed to the deep or superficial plane (Figures 1 and 2). There was no hypogastric mass, no palpable kidney mass, and no disorder of sex development. The penis was uncircumcised.

**Figure 3:**
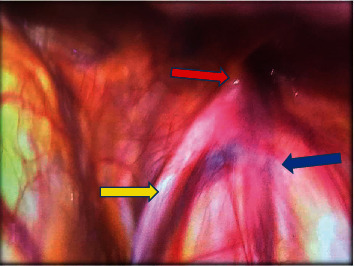
Laparoscopic view of the spermatic vessels (blue arrow) and vas deferens (yellow arrow) exiting the right “open” deep inguinal ring (red arrow).

**Figure 4:**
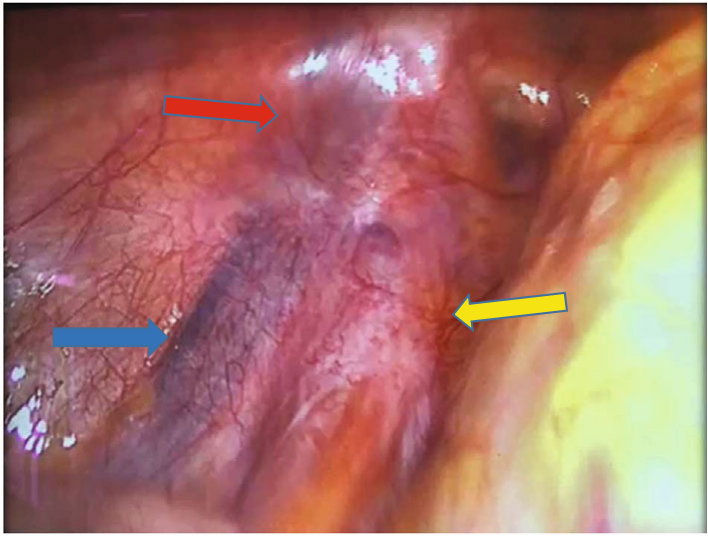
Laparoscopic view of the spermatic vessels (blue arrow) and vas deferens (yellow arrow) exiting the left “closed” deep inguinal ring (red arrow).

An abdominal ultrasound found no renal anomalies, and description of the left suprapubic mass was inconclusive. A diagnostic laparoscopy was planned to explore the right testis and any anomaly. On laparoscopic evaluation under general anesthesia, the right spermatic vessels and vas were seen going through an open right deep inguinal ring (Figure 3). The spermatic vessels and vas on the left were seen traversing their corresponding closed deep inguinal ring (Figure 4). There were no Müllerian remnants. To identify the origin of the two left-sided scrotal masses during laparoscopy, the lowermost intrascrotal mass was tugged and the effect was seen at the right deep inguinal ring. Meanwhile, tugging of the higher-placed suprascrotal mass resulted in denting of the left deep inguinal ring structures with no effect on the right deep inguinal ring.

**Figure 5:**
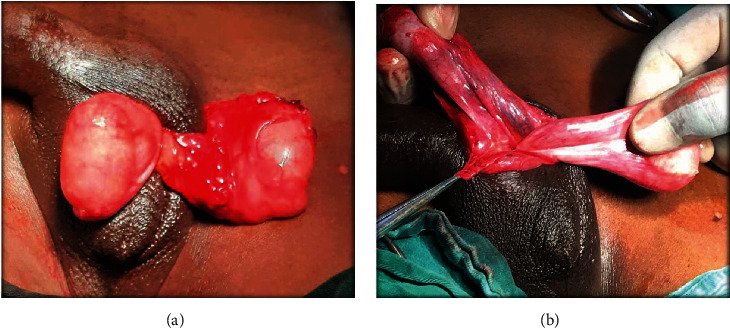
(a) Left and right testes shown through a median raphe scrototomy looking macroscopically normal. (b) No fusion of vessels nor vasa, nor epididymotesticular separation.

**Figure 6:**
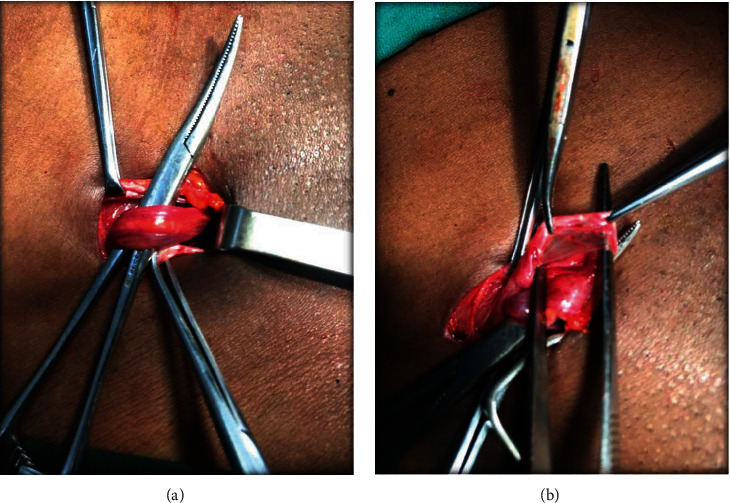
(a) Right inguinal dissection. (b) Closure of a right patent processus vaginalis.

**Figure 7:**
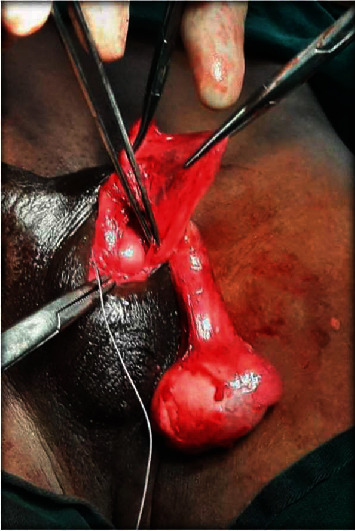
Transseptal orchidopexy of the right testis and closure of the scrotal septum.

**Figure 8:**
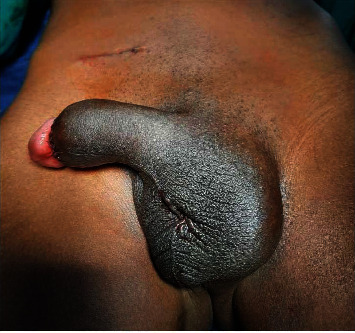
Final aspect after bilateral orchidopexy and routine circumcision.

**Figure 9:**
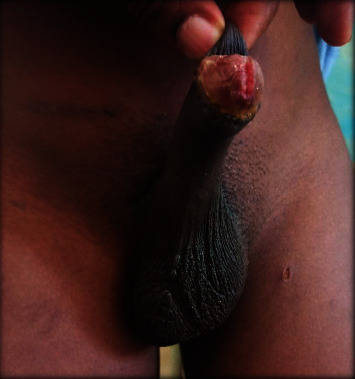
Scrotal aspect on day 5 postoperative.

A scrototomy through a median raphe incision was made. The lowermost testis was easily dissected and its cord traced to the right side passing front of the root of the penis. The higher placed testis' dissection was more laborious with adhesions and an inflammatory tunica. Both testis appeared macroscopically normal with no epididymotesticular fusion anomaly and unfused vasa deferentia (Figure 5). A right inguinal exploration found a patent processus vaginalis which was transected and closed at the deep ring. Transseptal orchidopexy was undertaken for the right testis with its longer cord length (Figure 6), and an ipsilateral orchidopexy was done for the nondescended suprascrotal left testis (Figure 7). Circumcision was done. The early postoperative period was uneventful with mild tenderness on palpation and a full-looking scrotum (Figures 8 and 9).

## 3. Discussion

Some theories have been postulated to explain this singular anomaly of testicular descent which vary depending on the presentation and associated anomalies. A faulty development of both testes on the same side was proposed by Lenhossek and later Berg [5]. Kimura [6] concluded that if both vasa deferentia arose from one side, there had been unilateral origin but if there was bilateral origin, one testis had crossed over due to some compression force. Gupta and Das [7] postulated that adherence and fusion of the developing Wolffian ducts took place early and that descent of one testis caused the second one to follow. This has been referred to as mechanical hindrance by Chacko and colleagues [8]. Defective implantation of the gubernaculum or an obstruction of the inguinal canal preventing testicular descent on the ipsilateral side was confirmed on rat models [9]. The presence of Müllerian structures in persistent Müllerian duct syndrome (PMDS) may bind to vasa and affect their descent [10, 11].

The spectrum of associated clinical findings most commonly includes ipsilateral inguinal hernia, hypospadias, PMDS, disorder of sex differentiation (DSD), fusion of vas deferens, seminal vesicle cysts, renal agenesis, and scrotal abnormalities [12, 13]. The classification proposed by Gauderer et al. [14] is based on the presence of associated abnormalities: Type 1, the most common type (40-50%) is associated with ipsilateral inguinal hernia alone; Type 2 (30%) is associated with persistent Müllerian duct structures; and Type 3 (20%) is associated with other genitourinary abnormalities without Müllerian remnants. We present a form of TTE wherein the spermatic cords go through their individual inguinal canals to end up in the same hemiscrotum. In our case, the right testis probably reached the contralateral scrotum first and acted as an obstacle to the normal descent of the left testis into its scrotal sac. A similar anatomic configuration has been described previously involving the left testicle [1].

The diagnosis was confirmed by laparoscopic visualization of cord structures passing through their separate deep inguinal rings, respectively. The left internal inguinal ring was closed but the right deep inguinal ring was open. This underscores the importance of laparoscopic exploration as a diagnostic modality to investigate undescended nonpalpable testes in place of other imaging like magnetic resonance imaging, ultrasonography, and computed tomography [15–17] which may describe some of the abnormalities associated with TTE.

The management goals are fixation of the testes, search for Müllerian duct remnants, and long-term follow-up for malignancy and fertility [18]. The overall incidence of malignant transformation of gonads is 18% [19]. There is no formal consensus on the management due to the rarity of the pathology and its varied presentation [20]. Nonetheless, algorithms based on extensive dissection of the cord [21] or none at all [22] have been proposed to guide management after intraoperative findings. Still, some authors advocate complete dissection to individualize the testes and spermatic cords with transseptal or extraperitoneal orchidopexy through an inguinal or scrotal approach [18]. Laparoscopic surgery with or without inguinal exploration has been described for management of TTE and associated anomalies in staged or single procedures [23, 24]. We used laparoscopy for diagnosis and then a scrotal approach to explore the left hemiscrotum and successfully placed both testes in separate scrotal compartments through a transseptal orchidopexy. There was no need for an ipsilateral inguinal incision because deep inguinal ring was closed on laparoscopic exploration. The follow-up will be ensured by regular examination of the testis through physical examination and ultrasound.

## 4. Conclusion

Nonpalpable testis in a child associated with contralateral full scrotum should lead to researching all potential sites of testicular ectopy. Laparoscopy should be privileged in the exploration as it can identify the paths of the different cords and avoid additional dissection. A new classification and consensus in the management of transverse testicular ectopy could be considered with regard to the variant form presented. In early or late presentation, a follow-up is warranted in terms of testicular size, functional status, and malignant transformation.
